# BETWEEN-PERSON AND DAILY WITHIN-PERSON VARIATION PATTERNS IN MEASURES OF VIEWS OF AGING

**DOI:** 10.1093/geroni/igad104.2695

**Published:** 2023-12-21

**Authors:** Erica O’Brien, Joshua Smyth

**Affiliations:** Pennsylvania State University, State College, Pennsylvania, United States; Pennsylvania State University, State College, Pennsylvania, United States

## Abstract

Despite increasing evidence of daily associations between individuals’ health and their age-related thoughts, beliefs, feelings and experiences (collectively referred to as views of aging, VOA), we know little about daily variation in VOA more generally. We used data from an online sample of 122 adults (aged 26 to 78 years) who completed multiple measures of VOA (subjective age, age group identity, aging attitudes, implicit theories of aging, awareness of age-related change) on each of 7 consecutive days, partitioning variance in responses to each measure at the person and day levels to assess between- and within-person variability, respectively. Results showed that, in all measures, between-person variability accounted for most of the total observed variation (62% to 92%) whereas within-person variability accounted for a significant but smaller amount (8% to 38%). The ratio of between- to within-person variation, however, depended on the specific measure and age group assessed, with the highest ratios observed for subjective age (versus other measures) and older adults (versus young adults). Findings suggest that VOA are relatively stable in daily life and may become more stable with advanced age. Further study of measures and age groups that exhibit the most within-person variability can yield insights that help increase understanding about the dynamics of VOA (i.e., which VOA constructs are most sensitive to fluctuating contexts and for whom?) and therefore inform future work linking VOA to other phenomenon in daily life.

